# Atorvastatin-Eluting Contact Lenses: Effects of Molecular Imprinting and Sterilization on Drug Loading and Release

**DOI:** 10.3390/pharmaceutics13050606

**Published:** 2021-04-22

**Authors:** Ana F. Pereira-da-Mota, María Vivero-Lopez, Ana Topete, Ana Paula Serro, Angel Concheiro, Carmen Alvarez-Lorenzo

**Affiliations:** 1 Departamento de Farmacología, Farmacia y Tecnología Farmacéutica, I+D Farma (GI-1645), Facultad de Farmacia and Health Research Institute of Santiago de Compostela (IDIS), Universidade de Santiago de Compostela, 15782 Santiago de Compostela, Spain; anafilipa.pereira@rai.usc.es (A.F.P.-d.-M.); mariavivero.lopez@usc.es (M.V.-L.); angel.concheiro@usc.es (A.C.); 2 Centro de Química Estrutural, Departamento de Engenharia Química, Instituto Superior Técnico, Universidade de Lisboa, Av. Rovisco Pais, 1049-001 Lisbon, Portugal; anatopete@tecnico.ulisboa.pt (A.T.); anapaula.serro@tecnico.ulisboa.pt (A.P.S.)

**Keywords:** atorvastatin, bioinspired contact lenses, molecularly imprinted hydrogels, computational modeling, sterilization, controlled drug release

## Abstract

Statins are receiving increasing attention in the ophthalmic field. Their activity as 3-hydroxy-3-methylglutaryl–CoA (HMG–CoA) reductase inhibitors is clinically used to regulate cholesterol levels and leads to pleiotropic effects, which may help in the management of diabetes-related ocular pathologies. This work aims to design bioinspired contact lenses (CLs) with an affinity for atorvastatin by mimicking the active site of HMG–CoA reductase. Sets of imprinted and nonimprinted 2-hydroxyethyl methacrylate (HEMA) hydrogels were synthesized, varying the contents in functional monomers that bear chemical groups that resemble those present in HMG–CoA reductase, namely, ethylene glycol phenyl ether methacrylate (EGPEM), 2-aminoethyl methacrylate hydrochloride (AEMA), and N-(3-aminopropyl) methacrylamide hydrochloride (APMA). The hydrogels were characterized in terms of suitability as CLs (solvent uptake, light transmission, mechanical properties, and biocompatibility) and capability to load and release atorvastatin. Three sterilization protocols (steam heat, gamma radiation, and high hydrostatic pressure) were implemented and their effects on hydrogel properties were evaluated. Copolymerization of AEMA and, particularly, APMA endowed the hydrogels with a high affinity for atorvastatin (up to 11 mg/g; K_N/W_ > 200). Only high hydrostatic pressure sterilization preserved atorvastatin stability and hydrogel performance. Permeability studies through the porcine cornea and sclera tissues revealed that the amount of atorvastatin accumulated in the cornea and sclera could be effective to treat ocular surface diseases.

## 1. Introduction

Statins, 3-hydroxy-3-methyl-glutaryl coenzyme A (HMG–CoA) reductase inhibitors, are a class of lipid-lowering drugs widely prescribed for regulation of cholesterol levels and notably contributing to the prevention of cardiovascular diseases [1]. They act on a key step of the metabolic pathway for the biosynthesis of intracellular cholesterol, i.e., the mevalonate pathway [2]. In addition to lowering cholesterol, statins are recognized to have pleiotropic effects, which are shown as anti-inflammatory, antiproliferative, neuroprotective activities, etc. [3]. Although there are not commercially available ophthalmic formulations of statins, oral administration has been associated with beneficial effects on eye conditions. With respect to ocular surface inflammation, preliminary data suggest that oral administration of statins may increase the regenerative quality of corneal repair promoting corneal wound healing [4]. Nevertheless, the effect of statins on dry eye symptoms and meibomian gland function is still unclear [5]. Statins cholesterol-lowering function may have a role in the prevention of cataracts since cholesterol synthesis is required to regulate the metabolism of the crystalline [6] and accumulation of cholesterol in human lens fibers facilitates cataractogenesis [7]. In open-angle glaucoma, statins may increase retinal and choroidal blood flow maintaining the optic nerve and retinal nerve fiber layer health, reducing the severity of glaucoma [8]. In age-related macular degeneration, statins may reduce drusen formation between the retinal pigment epithelium and Bruch membrane [9]. In diabetic retinopathy, statins appear to reduce hard exudates formation [10] and macular edema [11], improve the endothelial structure, function, blood flow velocity, and vascular resistance, reducing microaneurysm formation and leakage or hemorrhage from new vessels [12]. Overall, systemic delivery of statins is revealing beneficial to treat ophthalmic disorders affecting anterior and posterior segments. These therapeutic effects could be improved if efficient ocular formulations are developed.

Atorvastatin calcium is a synthetic statin with relatively moderate lipophilicity and low molecular weight, which may be able to penetrate the ocular structures if topically applied [13,14]. Preclinical studies have demonstrated that atorvastatin (50 µM) ophthalmic solutions have a greater immunomodulatory effect on activated human T cells than lovastatin and simvastatin solutions [15]. Moreover, atorvastatin drops can be safely administered for long-term treatment of dry eye and blepharitis, showing tear film stabilization and anti-inflammatory effects [16].

Diabetes mellitus is a metabolic disease defined by elevated blood glucose that leads to macrovascular (e.g., coronary heart disease, peripheral vascular disease, and stroke) and microvascular (e.g., retinopathy, nephropathy, and neuropathy) complications [17,18]. Dysregulation of blood glucose can cause alterations in a multitude of anterior and posterior structures of the eye [19]. The most well-studied manifestation is diabetic retinopathy, although it is estimated that diabetic keratopathy affects up to two-thirds of diabetic patients [17,20,21]. Diabetic keratopathy complications could be superficial punctate keratitis, corneal erosions, and persistent epithelial defects and, in advanced stages, loss of the basement membrane and subsequent stromal ulceration [19]. Corneal sensitivity is also impaired in diabetes and may predispose to bacterial corneal ulcers, compromising the healing mechanism [22]. Furthermore, tear film secretion and stability could be altered in diabetic patients [23,24]. Efficient wound healing mechanisms are important to maintain transparency and restore the barrier function of the cornea. The use of topical anti-inflammatory medications is useful for alleviating ocular surface inflammation and promoting re-epithelialization [20].

Contact lenses (CLs) are being positioned as advantageous platforms for the sustained release of ophthalmic drugs [25,26,27]. Once applied to the eye, the CL may release the drug toward the post-lens tear film, which has slower turnover than the lachrymal fluid in the absence of the lens. Prolonged permanence and higher drug levels on the ocular surface facilitate drug penetration into eye tissues [28,29,30]. Despite the therapeutic advantages of medicated CLs, their design is still a huge challenge because most available CLs lack affinity for ophthalmic drugs [31]. This problem can be overcome by means of bioinspired strategies that rely on mimicking the human pharmacological receptor of the drug into the CL structure [28]. The incorporation of functional monomers bearing chemical groups similar to those present in the natural drug binding site, and their arrangement assisted by molecular imprinting has been shown useful to improve the performance of CLs as platforms for the sustained release of acetazolamide and ethoxzolamide [32], ketotifen fumarate [30], olopatadine [33], epalrestat [34], naltrexone [35], transferulic acid [36], etc. This bioinspired strategy does not compromise the performance of CLs as medical devices for vision correction.

**Figure 1:**
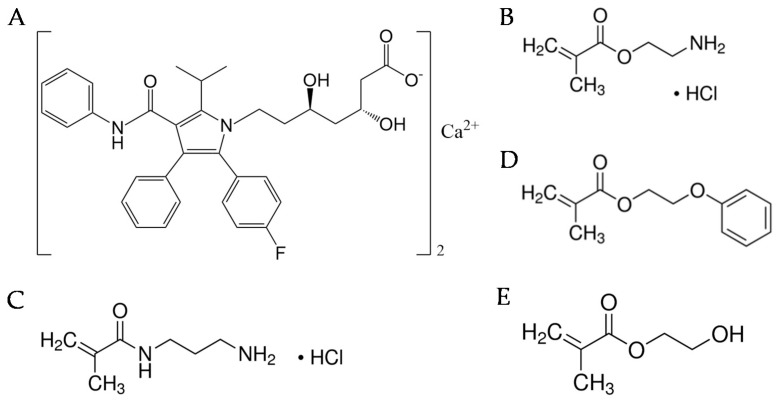
Structure of atorvastatin calcium (**A**) and the functional monomers used to synthesize the hydrogels: (**B**) 2-aminoethyl methacrylate hydrochloride (AEMA); (**C**) N-(3-aminopropyl) methacrylamide hydrochloride (APMA); and (**D**) ethylene glycol phenyl ether methacrylate (EGPEM), and (**E**) 2-hydroxyethyl methacrylate (HEMA).

The present work relies on the hypothesis that hydrogels that can mimic the components of the active site of HMG–CoA reductase might exhibit enhanced affinity for statins. Thus, the aim of the work was to design, for first time, atorvastatin-loaded CLs using functional monomers that bear chemical groups that resemble those present in HMG–CoA reductase and that can be successfully integrated with the CL network in order to optimize the loading and release of atorvastatin calcium while still preserving the properties required for CLs, mainly in terms of ocular tolerance, light transmission, and mechanical properties. Statins have a polar head group that mimics the natural substrate of HMG–CoA reductase and, thus, bind the active site, blocking the access of the natural substrate. In the binding of type II statins (as atorvastatin), the isopropyl group participates in Van der Waals contacts with the enzyme involving residues Leu^562^, Val^683^, Leu^853^, Ala^856^, and Leu^857^. Atorvastatin (Figure 1A) forms a hydrogen bond through its carbonyl oxygen atom with Ser^565^, while the fluorophenyl group can coordinate to Arg^590^ through polar interactions [37]. Taking into account the functionalities of the amino acids involved in the binding site of HMG–CoA reductase, ethylene glycol phenyl ether methacrylate (EGPEM), 2-aminoethyl methacrylate hydrochloride (AEMA), and N-(3-aminopropyl) methacrylamide hydrochloride (APMA) (Figure 1) were chosen as functional monomers after a first screening of the interaction with atorvastatin using computational modeling. Hydrogels were prepared from mixtures of 2-hydroxyethyl methacrylate (HEMA), EGPEM, AEMA, and APMA at various ratios (Table 1). Some hydrogels were synthesized in the presence of the drug (imprinted hydrogels) to verify whether the drug molecules could contribute to a better arrangement of the monomers for a more efficient formation of ad hoc artificial receptors.

Since sterilization is a mandatory step in the conditioning of CLs and may affect the material properties, the drug stability, and the drug release pattern, a relevant second aim of the present work was to evaluate to what extent several sterilization techniques may alter the capability of the designed CLs to act as platforms for atorvastatin release. The stability of the hydrogels and the drug in separate and of the drug-loaded hydrogels against steam heat (autoclave), gamma radiation, and high hydrostatic pressure (HHP) techniques was evaluated. Steam heat and gamma radiation are the most used methods for sterilization of medical devices [38]. Steam heat is efficient, fast, and does not involve toxic agents, but it is not suitable for materials and drugs that are heat sensitive [39]. Gamma radiation allows operating at low temperatures, but it is expensive, complex, and may affect the polymer properties [40]. HHP is increasingly used in the food industry to inactivate microorganisms without altering the food properties [41,42]. Pressure-based methods are also used for the disinfection of biomaterials and for vaccine production [42]. Furthermore, according to previous reports [43], HHP seems to be an alternative method for terminal sterilization of drug-loaded intraocular lenses.

Finally, the most promising CLs were evaluated regarding cytocompatibility with Balb/3T3 fibroblasts, ocular tolerance (HET–CAM), and ex vivo accumulation/permeability of atorvastatin into/through the porcine cornea and sclera tissues.

## 2. Materials and Methods

### 2.1. Materials

Atorvastatin calcium was from Biosynth (Compton, UK). 2-Hydroxyethyl methacrylate (HEMA) was supplied by Merck (Darmstadt, Germany). Ethylene glycol dimethacrylate (EGDMA), ethylene glycol phenyl ether methacrylate (EGPEM), 2,2′-azo-bis(isobutyronitrile) (AIBN), 2-aminoethyl methacrylate hydrochloride (AEMA), and dichlorodimethylsilane were from Sigma-Aldrich (Steinheim, Germany). N-(3-aminopropyl) methacrylamide hydrochloride (APMA) was from PolySciences Inc. (Warrington, PA, USA). Sodium chloride (NaCl) was from Scharlau (Barcelona, Spain), and ethanol absolute was from VWR Chemicals (Leuven, Belgium). Ultrapure water (resistivity > 18.2 MΩ cm; Milli-Q^®^, Millipore Ibérica, Madrid, Spain) was obtained by reverse osmosis. Simulated lachrymal fluid (SLF) was prepared with the following composition in water: 6.78 g/L NaCl from Scharlau (Barcelona, Spain), 1.90 g/L NaHCO_3_ from Probus (Barcelona, Spain), 1.38 g/L KCl, and 0.042 g/L CaCl_2_ from Merck (Darmstadt, Germany) with pH 7.4 [23]. Carbonate buffer pH 7.2 was prepared by mixing two buffer solutions: buffer solution A (100 mL: 1.24 g NaCl, 0.071 g KCl, 0.02 g NaH_2_PO_4_ from Merck (Darmstadt, Germany), 0.49 g NaHCO_3_) and buffer solution B (100 mL: 0.023 g CaCl_2_, 0.031 g MgCl_2_ from Panreac (Barcelona, Spain)). Balb/3T3 fibroblasts were provided by American Type Culture Collection (Manassas, VA, USA).

### 2.2. Computational Modeling

The 3D structure of the functional monomers (EGPEM, AEMA, and APMA) and atorvastatin calcium was taken from the PubChem database [44]. The Autodock Tools version 4.2.6 software (Molecular Graphics Lab., La Jolla, CA, USA) was used to perform computational molecular docking in two steps, using a semiempirical free energy force field. The molecules under study, the ligand (in this case, the monomer) and the receptor (drug) started in an unbound conformation, and after that, the intramolecular energetics were estimated from these unbound states to the conformation in the bound state. Then, the intermolecular energetics of their bound conformation were analyzed using the Lamarckian genetic algorithm [45]. Finally, estimated free energy of binding (∆G_binding_) and dissociation constant (Ki) values were obtained [46]. In all cases, the grid was generated with default settings around the monomer and the drug.

### 2.3. Hydrogel Preparation

Compositions of the monomers’ mixtures and the amounts of atorvastatin calcium added are shown in Table 1. The hydrogels were designed with AF codes, followed by a number referring to a specific combination of monomers and one/two letters, which indicate whether the networks were imprinted (letter i) or not (letters ni). The components were mixed under magnetic stirring (400 rpm) for 120 min and then 5 min in sonication. AIBN (initiator) was added, and the solutions were magnetically stirred again (100 rpm) for 10 min. Finally, the solutions were injected into molds made from two glass plates (10 × 10 cm) previously treated with dichlorodimethylsilane and separated by a silicone frame (0.3 mm thickness) [47]. The polymerization was carried out by heating at 50 °C for 12 h, then the temperature was raised to 70 °C, and the polymerization was continued for a further 24 h. In such a way controlled thermal decomposition of the initiator AIBN prevented nitrogen bubbles formation [48].

After polymerization, each hydrogel was immersed in boiling water (1000 mL) for 15 min to remove unreacted monomers and to facilitate the cut as 10 mm discs. The hydrogels were washed in ultrapure water for 24 h replacing the medium twice a day and then 0.9% NaCl and in water, every other day, replacing the medium twice a day for 10 days. During the cleaning process, the medium was kept under magnetic stirring (200 rpm) at room temperature and protected from the light. The cleaning process was monitored spectrophotometrically (Agilent 8453, Waldbronn, Germany) for both residual monomers leakage (only observed in the first washing solutions) and quantification of the amount of atorvastatin extracted. Atorvastatin was quantified from absorbance measurements at 242 nm using a validated calibration curve prepared for drug solutions in ethanol:water (20:80 *v/v*). Finally, the discs were dried at 70 °C for 24 h.

### 2.4. Hydrogel Characterization

Solvent uptake. Dried hydrogel discs were weighed (W_0_) and immersed in SLF or atorvastatin calcium solution (0.04 mg/mL in ethanol:water 20:80 *v/v*) for 24 h at room temperature. Four replicates were tested for each hydrogel type. At a pre-established time (0.5, 1, 1.5, 2, 3, 4, 5, 6, and 24 h), the discs were removed, carefully wiped, and weighed (W_t_). The solvent uptake was monitored as the increase in weight with respect to the dried disc as the following equation:

Solvent uptake (%) = [(W_0_ − W_t_)/W_0_] × 100 (1)

Light transmission. The transmittance of discs hydrated in SLF and atorvastatin calcium solution (0.04 mg/mL in ethanol:water 20:80 *v/v*) was recorded, in quadruplicate, in 200–800 nm range (UV–Vis spectrophotometer, Agilent 8453, Boeblingen, Germany). 

Mechanical properties. The mechanical properties of hydrated hydrogels (in 0.9% NaCl) were evaluated through tensile tests using a TA.XT Express Texture Analyser (Stable Micro Systems, Godalming, UK), with a 0.005 N trigger force, 50 N load cell, and test speed of 0.25 mm/s. Each hydrogel was cut with a dog bone-shaped mold (total 18 × 5 mm, in the center 6 × 2.5 mm). The samples were fixed to the upper and lower clamps. The Young’s modulus was determined from the initial slope of the obtained stress–strain curves. Five independent experiments were conducted for each hydrogel formulation, before and after the different sterilization methods.

### 2.5. Atorvastatin Loading and Release

Nonimprinted and imprinted dried discs, in quadruplicate, were weighed (approx. 20 mg) and immersed in vials containing 10 mL of atorvastatin calcium solution (0.04 mg/mL in ethanol:water 20:80 *v/v*). The vials were kept under magnetic stirring (150 rpm) at room temperature and protected from the light for 48 h. At pre-established periods of time, the absorbance of the loading solution was measured by UV spectrophotometry (Agilent 8453, Boeblingen, Germany) at 242 nm and the amount of atorvastatin loaded was calculated as the difference between the initial and final amount of drug in the solution. The network/water partition coefficient of atorvastatin, K_N/W_, was estimated for each hydrogel type from the total amount loaded as follows [49]:

Amount loaded = [(V_s_ + K_N/W_ × V_p_)/W_p_] × C_0_ (2)

In this equation, V_s_ is the volume of solution absorbed by the hydrogel, V_p_ the dried polymer volume, W_p_ the dried hydrogel weight, and C_0_ the drug concentration in the loading solution.

Atorvastatin-loaded discs were rinsed with SLF and the discs were placed in vials with 10 mL of SLF (pH = 7.4, similar to the precorneal film [50]) at 37 °C and magnetically stirred at 150 rpm. The vials were protected from the light. Samples of the medium (3 mL) were periodically withdrawn and returned to the vial immediately after absorbance measurement at 242 nm. After 8 h and until the end of the test, samples of the release medium (2 mL) were withdrawn and replaced with the same volume of fresh SLF to maintain sink conditions and avoid false plateaus.

### 2.6. HET–CAM Test

The hen’s egg test chorioallantoic membrane (HET–CAM) assay was used for preliminary screening of ocular irritancy of the hydrogels according to the ICCVAM protocol [51], as previously described [34]. Briefly, fertilized hen’s eggs (Coren, San Cibrao das Viñas, Spain) were incubated at 37 °C with 60% relative humidity for 7 days and were manually rotated 180° three times a day for the duration of the incubation. On the eighth day, the eggs were cut on the wider extreme to extract the eggshell, remove the inner membrane, and expose the chorioallantoic membrane (CAM). Atorvastatin-loaded hydrogel discs and 300 µL of atorvastatin calcium (0.04 mg/mL in ethanol:water 20:80 *v/v*) were carefully placed on the CAM and the possible hemorrhage, vascular lysis, or coagulation of the vessels were monitored for 5 min. Two independent experiments were carried out for each hydrogel and the drug solution. The discs were previously loaded with atorvastatin calcium for 48 h, as described above. NaCl (0.9%) and NaOH (0.1 N) solutions were used as negative and positive controls, respectively. Then, the irritation score (IS) was estimated, as previously reported [35].

### 2.7. Sterilization

Three different sterilization protocols were tested for hydrogels previously swollen in NaCl 0.9% for at least 24 h or in drug solution (0.04 mg/mL) for 48 h and maintained in the same solutions: (i) steam heat (SH) sterilization for 1 h using an autoclave (Uniclave 88/75L; A.J. Costa, Cacém, Portugal) at 121 °C and 1 bar [43,52]; (ii) gamma-radiation (GI) sterilization with 25 kGy radiation dose [53] and a dose rate of ≈5 kGy/h at room temperature (only for the hydrogels without drug); and (iii) high hydrostatic pressure (HHP) at 70 °C and 600 MPa for 10 min [43]. Four independent experiments were carried out for each hydrogel and sterilization condition. For the HHP, each hydrogel disc was previously packed and sealed in polyamide/polyethylene vacuum bags filled with NaCl 0.9% or drug solution and preheated at 70 °C for 10 min. Then, the bags were placed inside a polytetrafluoroethylene insulation vessel prefilled with water at the same temperature and immediately pressurized in a Hiperbaric 55 equipment (Burgos, Spain) [54].

Aliquots of the drug solution (0.02 mg/mL in ethanol:water 20:80 *v/v*) were sterilized under the same conditions as the hydrogels and thereafter analyzed by UV–Vis spectrophotometry and HPLC (as described in Section 2.10).

After the sterilization processes, the mechanical properties of fully hydrated hydrogels (in NaCl 0.9%) previously equilibrated at room temperature were evaluated. Furthermore, the loading and release experiments were carried out again with the discs sterilized in NaCl 0.9% medium and that were washed in water for 24 h and dried at 70 °C for another 24 h under the same conditions as described above (Section 2.5).

### 2.8. FTIR–ATR Analysis

The FTIR-attenuated total reflectance (ATR) spectra of the nonsterilized and sterilized AF1, AF6, AF6ni, and AF6i hydrogels (after steam heat, gamma radiation, and high hydrostatic pressure) were recorded in an FTIR Varian 670-IR equipped with a PIKE GladiATR Diamond Crystal Attenuated Total Reflectance accessory with a resolution of 4 cm^−1^ and 64 scans.

### 2.9. Cytocompatibility Studies

Nonloaded and loaded hydrogels (AF6, AF6ni, and AF6i) were evaluated against Balb/3T3 fibroblasts cells (ATCC CCL-163; Manassas, VA, USA). Hydrogels (10 mm in diameter; approx. 20 mg) were loaded for 48 h in an atorvastatin solution (0.04 mg/mL in ethanol:water 20:80 *v/v*) or immersed in 0.9% NaCl solution (nonloaded hydrogels), cut in four symmetric pieces, and sterilized by HHP at 70 °C and 600 MPa for 10 min. Balb/3T3 cells were cultured in T75 flasks using Dulbecco’s modified Eagle medium (DMEM; Fisher Scientific, Newington, NH, USA) supplemented with 10% fetal bovine serum and 1% antibiotics (10,000 U/mL penicillin and 10,000 µg/mL streptomycin) in an incubator at 37 °C and 5% CO_2_. Then, cells were detached from the flasks at 80% confluence using TrypLE^®^ (Sigma Aldrich, St. Louis, MO, USA). Suspended cells were counted, seeded in wells of 96-well plates (20,000 cells/well), and allowed to attach for 24 h. Pieces (five) of discs were then placed individually in contact with the cells incubated for 24 h. Cells incubated in culture medium (negative control) and cells incubated in medium containing atorvastatin (0.04 and 0.02 mg/mL) were also evaluated.

The viability of the cells was evaluated using a Cell Counting kit-8 (CCK-8; Dojindo Molecular Technologies, Rockville, MD, USA) following the instructions from the manufacturer. Briefly, samples and culture medium were removed from the wells and 200 µL of a CCK-8 working solution (10 % *v/v* CCK-8 reagent in complete culture medium) were added to each well and incubated for 1 h at 37 °C. Finally, absorbance was measured at 450 nm (UV Bio-Rad Model 680 microplate reader, Hercules, CA, USA), and cell viability (%) was calculated as follows.

Cell viability (%) = (Abs _exp_/Abs _negative control_) × 100 (3)

### 2.10. Cornea and Sclera Permeability and Accumulation

Ex vivo permeability assays were carried out, in triplicate, with nonsterilized AF6, AF6ni, and AF6i loaded hydrogels following the BCOP test protocol, as previously described [55,56]. Porcine eyes were collected from a local slaughterhouse in the first hour after death and transported completely immersed in PBS solution in an ice bath. Corneas and scleras were isolated using a scalpel, washed with PBS, and mounted in vertical diffusion (Franz) cells. The donor and receptor chambers were filled with carbonate buffer pH 7.2 and maintained in a bath at 37 °C, gentle magnetic stirring. After 30 min, the solution in the donor chamber was completely removed using a Pasteur pipette and the corneas and scleras were exposed for 6 h to atorvastatin-loaded discs (AF6, AF6ni, and AF6i discs with 2 mL of SLF) or to control atorvastatin solution (60 µg/mL in ethanol:water 20:80 *v/v*, 1 mL). The donor chambers were covered with parafilm to prevent evaporation. Samples (1 mL) were removed from the receptor chamber at 0.5, 1, 2, 3, 4, 5, and 6 h, replacing with the same volume of fresh carbonate buffer each time and taking care of removing air bubbles from the diffusion cell.

The amount of atorvastatin in the receptor medium samples was quantified by HPLC (AS-4140 Autosampler, PU-4180 Pump, LC-NetII/ADC Interface Box, CO-4060 Column Oven, MD-4010 Photodiode Array Detector, JASCO, Tokyo, Japan) fitted with a C18 column (Waters Symmetry C18, 5 µm, 4.6 × 250 mm) and operated with ChromNAV software v.2. Mobile phase consisted of methanol: 0.05 M sodium phosphate (NaH_2_PO_4_) buffer (70:30 *v/v*, pH adjusted to 4.1 with o-phosphoric acid) at 1.00 mL/min and 25 °C [57]. The injection volume was 50 µL, and the total run time of each sample was 10 min. Atorvastatin was quantified at 247 nm (retention time of 6.3 min). The HPLC method was validated using two different calibration curves of atorvastatin in carbonate buffer, one between 0.125–2 µg/mL and the other one 2–16 µg/mL. The limit of detection (LOD) and limit of quantification (LOQ) values were 0.05 and 0.125 µg/mL, respectively.

After 6 h of experiment, the discs and solutions were removed from the donor chambers. The corneas and scleras were also removed from the diffusion cells and rinsed with 0.9% NaCl and immersed in 3 mL of ethanol:water (50:50 *v/v*) overnight at 37 °C under magnetic stirring. Then, they were sonicated for 99 min at 37 °C, centrifuged (1000 rpm, 5 min, 25 °C), and the supernatant filtered (Scharlau^®^ Syringe Filter, 0.22 µm 13 mm PTFE hydrophilic), centrifuged again (14,000 rpm, 20 min, 25 °C), and filtered to be measured by HPLC, as explained above.

### 2.11. Light Stability of Atorvastatin Calcium Solution

Solutions of atorvastatin calcium (0.02 mg/mL in ethanol:water 20:80 *v/v*) were placed in quartz cells (2 mL per cell) and exposed to fluorescent tube light (F8T5 Daylight, 6500 K, HITACHI) for 8 h at room temperature. The experiments were carried out in duplicate. UV–Vis spectra and HPLC chromatograms were recorded before and after exposition.

### 2.12. Statistical Analysis

Statistical analysis was performed using SPSS v.26.0 (IBM Co., Armonk, NY, USA). The descriptive data are presented as mean ± standard deviation. The normality of all variables was evaluated using the Shapiro–Wilk test. If normality was respected, a paired-samples *t*-test was used to compare the means between the two groups. One-way analysis of variance was used to compare the means of more than two groups. If normality was not verified, nonparametric tests were performed using Kruskal−Wallis test with post hoc Games–Howell correction. The level of significance used was 0.05.

## 3. Results and Discussion

### 3.1. Hydrogels Synthesis and Conditioning

**Figure 2:**
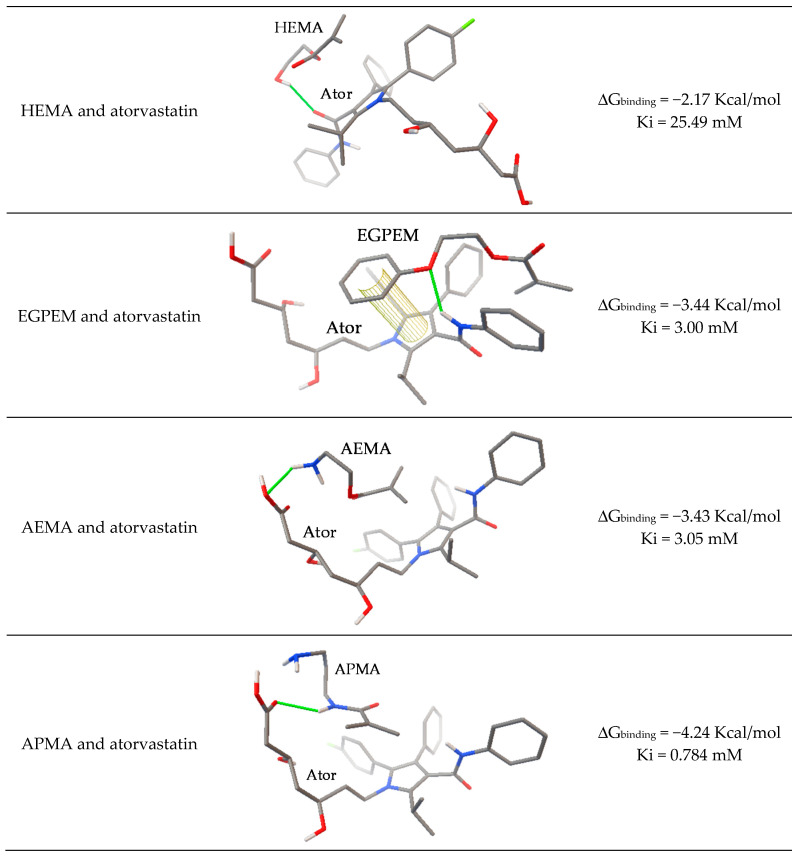
AutoDock modeling of structural and functional monomers–atorvastatin (Ator) interactions and the respective estimated free energy of the binding and inhibition constant. In the structures, N atoms are shown in blue, oxygen atoms in red, and the main bond interaction is shown in green.

The intensity of the interactions between atorvastatin calcium and the functional monomers was preliminary assessed using computer-assisted molecular modeling, which is considered as a green screening strategy for the rational selection of the composition [58,59]. The estimated free energy of the binding, inhibition constant, and the most probable structure are shown in Figure 2. Atorvastatin interacted with the structural monomer (HEMA) through a hydrogen bond between the hydrogen atom of the hydroxyl group and the carbonyl group of atorvastatin. Atorvastatin interacted with EGPEM through a hydrogen bond between the N atom of the amido group of atorvastatin and the oxygen ether group of EGPEM, stabilized through π–π stacking of the benzyl groups of both molecules. In the case of AEMA and APMA, the interaction occurred between the amino group of these monomers and the carboxylic acid group of atorvastatin. The interaction of the drug with APMA was remarkably more favorable both in terms of binding energy (∆G_binding_) and stability against dissociation (Ki). The interaction with the two other monomers, EGPEM and AEMA, was thermodynamically weaker but still favorable. Therefore, the three functional monomers were investigated regarding their capability to endow the hydrogels with an affinity for atorvastatin. It should be noted that the ∆G_binding_ values, although small, are in the same order of magnitude as those reported for other functional monomer:template 1:1 interactions using similar computational modeling software [60,61]. The binding enthalpy reported for HMG–CoA reductase and atorvastatin calcium in microcalorimetry titration experiments was higher (−10.9 Kcal/mol) but still in a comparable range [62].

Sets of hydrogels were prepared by combining AEMA, APMA, and EGPEM at various proportions as summarized in Table 1. AF1, AF1ni, and AF1i hydrogels were prepared without any functional monomer to be used as control. The first set of hydrogels AF2–AF6 was prepared with a concentration of APMA or AEMA of 40 mM and EGPEM of 200 mM. All monomers dissolved rapidly in HEMA medium with the exception of AEMA, and therefore AF3 and AF5 did not incorporate all feed AEMA content. Therefore, the second set of nonimprinted (AF1ni–AF6ni) and imprinted (AF1i–AF6i) hydrogels were prepared with lower concentrations of functional monomers (30 mM). Atorvastatin dissolved easily in the monomer solutions. Atorvastatin amount corresponded to drug:functional monomer 1:4 molar ratio. After polymerization, the hydrogel sheets were boiled in distilled water, which is the typical cleaning process of soft CLs after fabrication. Then, the hydrogels were successively immersed in water and NaCl 0.9% for the complete removal of atorvastatin before loading assay. Cleaning with NaCl 0.9% solution was needed to weaken the ionic interactions of atorvastatin with the hydrogels prepared with AEMA and APMA, as previously observed for other HEMA-based hydrogels [63]. The removal process was monitored spectrophotometrically at 242 nm until no leakage of drug or monomers was recorded.

### 3.2. Hydrogels Characterization

**Figure 3:**
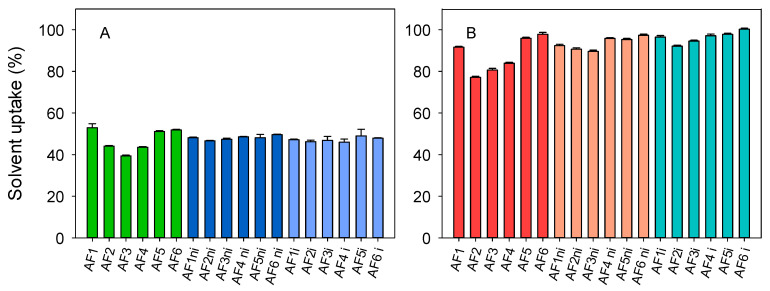
Solvent uptake (%) in (**A**) simulated lachrymal fluid and (**B**) atorvastatin calcium solution (0.04 mg/mL in ethanol:water 20:80 *v/v*) for all hydrogels before sterilization. Codes as in Table 1 (*n* = 4; mean values and standard deviations).

Dried discs rapidly sorbed SLF (Figure 3) and the equilibrium was reached in one hour. The hydrogels prepared with the highest amount of EGPEM (AF2, AF3, and AF4) presented the lowest solvent uptake either in SLF or atorvastatin calcium solution because of the hydrophobicity of this monomer. Imprinted and nonimprinted hydrogels presented similar solvent uptake values. The solvent uptake in atorvastatin solution was significantly higher than that recorded in SLF, about 90% due to the addition of ethanol. Organic solvents as ethanol increase the solubility of the hydrophobic moieties of PHEMA in the aqueous medium, promoting hydrogel swelling [64]. Regarding light transmission, all hydrated hydrogels were transparent in the visible range (400–700 nm), with transmittance values close to or above 90% (Figure S1 in Supplementary Materials) [65]. The presence of the drug did not alter the transmittance in the visible region.

### 3.3. Atorvastatin Loading and Release

**Figure 4:**
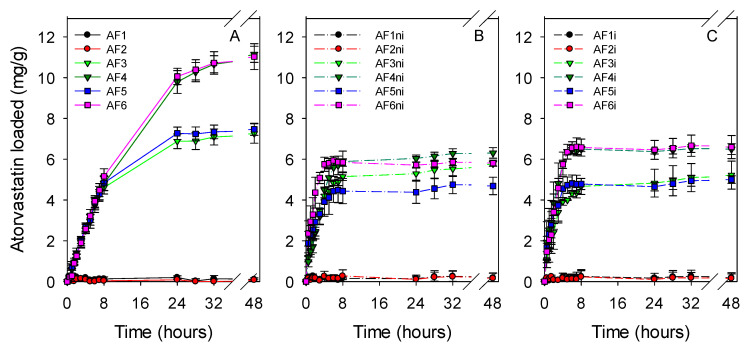
Atorvastatin loading profiles of all hydrogels ((**A**) first set of hydrogels; (**B**) nonimprinted hydrogels and (**C**) imprinted hydrogels) soaked in 10 mL of atorvastatin solution (0.04 mg/mL in ethanol:water 20:80 *v/v*) at room temperature under magnetic stirring (150 rpm) for 48 h. Hydrogel codes as in Table 1 (*n* = 4; mean values and standard deviations).

Hydrogel discs were dried and immersed in atorvastatin solution (0.04 mg/mL in ethanol:water 20:80 *v/v*) to quantify the drug loading ability (Figure 4). Nonfunctionalized hydrogels did not uptake any atorvastatin (AF1, AF1ni, and AF1i). Predictably, the amount of atorvastatin loaded increased with the content in APMA and AEMA functional monomers. The hydrogels with larger content in functional monomers required more than 24 h to complete the sorption, while the other nonimprinted and imprinted hydrogels completed the loading in the first 8 h. Addition of EGPEM did not further improve the loading (code AF2). The uptake was remarkably higher for hydrogels prepared with APMA (AF4, AF6, AF4ni, AF6ni, AF4i, and AF6i), which had the highest affinity for atorvastatin. Imprinted hydrogels showed minor increases in the amount of atorvastatin loaded compared to nonimprinted ones, which is typical of drugs in which one-point interaction prevails over other feasible interactions [66].

Compared to nonfunctionalized hydrogel (AF1) and EGPEM hydrogels (AF2), hydrogels bearing AEMA or APMA hydrogels showed two orders of magnitude greater K_N/W_ values (Table 2). The highest K_N/W_ values were recorded for hydrogels with the largest content in functional monomers. APMA hydrogels (AF4 and AF6) had statically significant higher (*p* < 0.05) K_N/W_ values than AEMA hydrogels (AF3 and AF5), in good agreement with the computational modeling analysis. Comparing the imprinted and nonimprinted hydrogels, no statistically significant differences in K_N/W_ values were recorded, except for AF6ni and AF6i (*p* < 0.05). The K_N/W_ values of atorvastatin recorded for hydrogels prepared with AEMA or APMA were higher than those previously found for functionalized CLs designed to uptake other drugs such as naltrexone [35], polymyxin B [67], acetazolamide, ethoxzolamide [32], or ferulic acid [36]. High K_N/W_ values can be typically found for drugs that can establish strong ionic interactions with the hydrogel moieties, as observed for epalrestat and APMA-functionalized networks [34].

**Figure 5:**
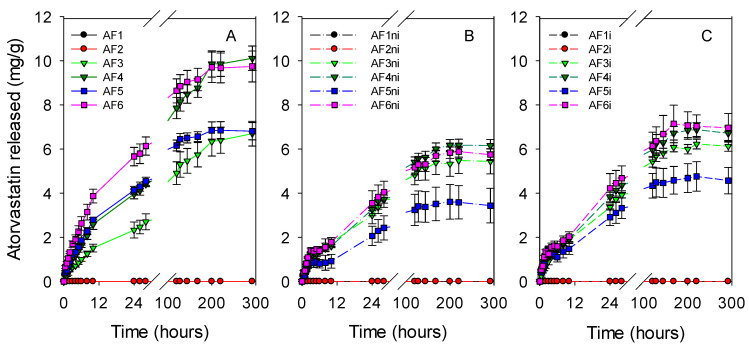
Atorvastatin release profiles from drug-loaded discs ((**A**) first set of hydrogels; (**B**) nonimprinted hydrogels and (**C**) imprinted hydrogels) in SLF at 37 °C under magnetic stirring (150 rpm). Codes as in Table 1 (*n* = 4; mean values and standard deviations).

Atorvastatin-loaded discs were rinsed with water and immersed in 10 mL of SLF at 37 °C (sink conditions). The vials were maintained under oscillatory movement (150 rpm) to avoid the formation of a boundary layer around the discs, which could delay the release and lead to false sustained release [68]. The hydrogels prepared with the largest contents in functional monomers showed release patterns (Figure 5) similar to those recorded for less functionalized hydrogels when plotted as percentage released (Figure S2 in Supplementary Materials). Relevantly, all hydrogels provided sustained release almost for one week, covering extended wear of CL. The Higuchi equation was fitted quite well to the release profiles, indicating that drug diffusion through the swollen network is the main mechanism regulating the release [34] (Table S1 in Supplementary Materials).

### 3.4. HET–CAM Test and Cytocompatibility

The hen’s egg test on the chorioallantoic membrane is a fast and sensitive procedure that provides useful preliminary information about ocular irritancy and may be a validated alternative to the low volume eye test scale developed for rabbits [69]. The CAM tissue of fertilized hen eggs is noninnervated and contains arteries, veins, and capillaries that respond to injury in an analog way to ocular conjunctiva [70]. Images of CAM after 5 min exposure to atorvastatin calcium solution (300 µL, 0.04 mg/mL in ethanol:water 20:80 *v/v*) and atorvastatin-loaded discs showed no damages, as occurred for the negative control (NaCl 0.9%) (Figure S3 in Supplementary Materials). The presence of small contents of ethanol in the drug loading solution and in the atorvastatin-loaded hydrogels did not cause irritation, as previously reported for formulations containing up to 33% ethanol [71]. Differently, the positive control (NaOH 0.1 N) caused an irritation score (IS) of 18.82. Therefore, all formulations can be considered as nonirritant for the ocular surface.

**Figure 6:**
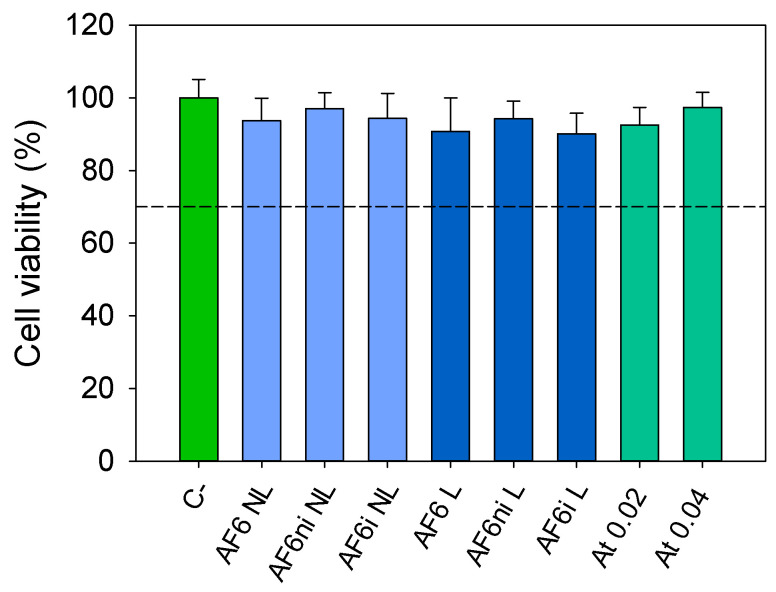
Viability of Balb/3T3 cells (%; mean and standard deviations) determined by CCK-8 assay, after 24 h exposure to nonloaded (NL), and atorvastatin loaded (L) hydrogels (AF6, AF6ni and AF6i) and two atorvastatin solutions (At; 0.04 and 0.02 mg/mL in ethanol:water 20:80 *v/v*). Negative control (C-) cells cultured in the absence of any treatment. Dashed line corresponds to 70% cell viability.

The cytotoxicity of the sterilized hydrogels (nonloaded and loaded with atorvastatin) was assessed through exposure to Balb/3T3 cells (Figure 6). The loading solution (0.04 mg/mL) and a solution containing the maximum amount of atorvastatin loaded by the hydrogels after 48 h (0.02 mg/mL) were also evaluated. In all cases, cell viability was above 90%. Overall, the designed hydrogels did not produce cytotoxic effects and could be safely used as biomaterials suitable for contact lenses, according to ISO 10993-5:2009 [72].

### 3.5. Effects of Sterilization

**Figure 7:**
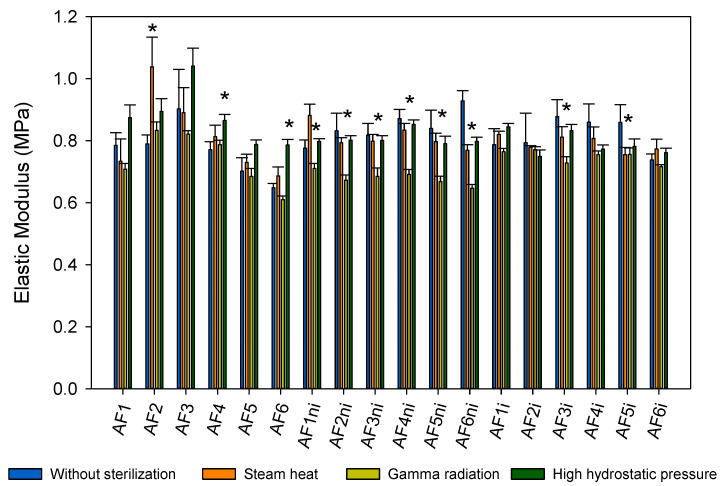
Elastic modulus (MPa) obtained for all hydrogels hydrated with 0.9% NaCl before and after each sterilization method. (*) Statistically different from the nonsterilized hydrogels (Kruskal–Wallis test and Games–Howell post hoc test, *p* < 0.05).

Sterilization of CLs is usually carried out by steam heat (autoclave) or gamma radiation [73]. Sterilization becomes a big challenge for drug-loaded CLs because it may compromise the stability of the drug or impact negatively on the drug release profile [43]. In the present work, steam heat, gamma radiation, and HHP were tested in order to evaluate their effects on the material properties and on their subsequent capability to load atorvastatin and control drug release. First, hydrogels were sterilized while immersed in a NaCl solution, and the elastic modulus was evaluated (Figure 7). Steam heat sterilization did not significantly affect the elastic modulus of any hydrogel (Kruskal–Wallis test, *p* > 0.05). Differently, gamma radiation significantly decreased the elastic modulus value of AF1ni, AF2ni, AF3ni, AF4ni, AF5ni, AF6ni, AF3i, and AF5i hydrogels (Kruskal–Wallis test, *p* < 0.05). HHP sterilization increased significantly the elastic modulus to 0.87 ± 0.02 MPa for AF4 and 0.79 ± 0.0 for AF6 (Kruskal–Wallis test, *p* < 0.05). Nevertheless, in all cases, the changes were small in magnitude and the elastic modulus obtained of the sterilized hydrogels (0.61–1.04 MPa) remained within the typical values for SCLs (0.38–1.44 MPa) [74].

To gain further insight into the effects of the sterilization technique on the hydrogel structure, the FTIR–ATR spectra of AF1, AF6, AF6ni, and AF6i dried discs before and after SH and HPP sterilization were compared (Figure S4 in Supplementary Materials). The spectra were normalized taking as a reference the main absorption peak of HEMA at 1704 cm^−1^, corresponding to the C=O band [75]. AF1, which lacks functional monomers, showed the typical bands of pHEMA networks with strong intensities at 1483 (CH_2_), 1367 (CH_2_), 1153, and 1073 (C–O–C) cm^−1^. Relevantly, none band of AF1 was altered after sterilization by either steam heat or HHP. The spectra of nonsterilized AF6, AF6ni, and AF6i evidenced a relative increase in the bands at 1367, 1153, 1073, and 965–938 cm^−1^, which can be attributed to the copolymerization with APMA. Relevantly, the sterilization did not cause significant changes in the spectrum bands with respect to the nonsterilized discs. Unfortunately, FTIR spectra were not useful to analyze atorvastatin–hydrogel interactions probably because of the relatively low content in the drug.

Concerning the drug, as a first step, the effect of the different sterilization processes on the atorvastatin stability was evaluated. The UV–Vis spectra of the drug solutions acquired before and after steam heat (121 °C, 1 bar for 1 h) and HHP sterilization (600 MPa, 10 min, and 70 °C) (Figure S5 in Supplementary Materials) were quite similar to that of nonprocessed solution. A strong decrease in the absorbance was recorded after gamma-radiation (25 kGy), indicating that the drug suffered degradation. Previous studies have shown that many drugs (e.g., moxifloxacin, ketorolac, diclofenac) degrade when exposed to gamma radiation in solution [43] due to the presence of free radicals and ions formed during the radiolysis of water [76]. Atorvastatin stability during steam heat and HHP was further investigated by HPLC (Figure S6 in Supplementary Materials). The drug was found to be sensitive to 121 °C originating degradation products with retention times of 3.62, 5.98, and 6.85 min. Goel et al. also reported atorvastatin degradation when heating the solution at 80 °C for 4 h [77]. Contrarily, HHP, which involved heating at 70 °C for 10 min and pressurize with 600 MPa did not trigger degradation.

**Figure 8:**
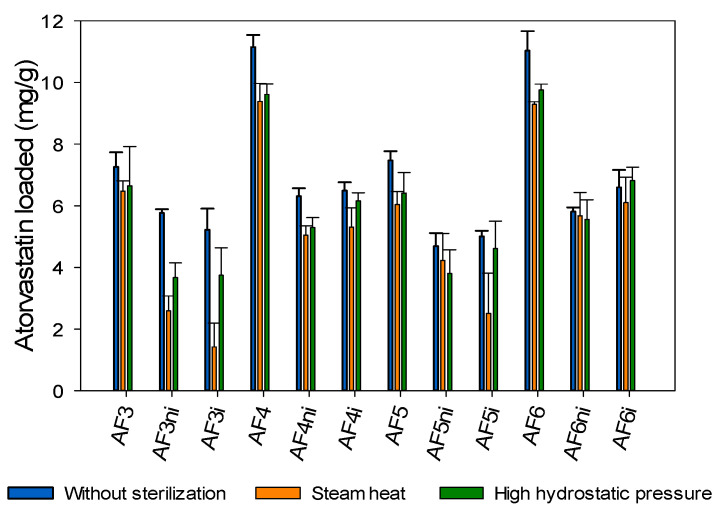
Atorvastatin loaded, after 48 h soaking, by hydrogels that had not been sterilized (blue bars) and hydrogels sterilized by steam heat (orange bars) or high hydrostatic pressure (green bars) before the loading. Codes as in Table 1 (*n* = 4; mean values and standard deviations).

**Figure 9:**
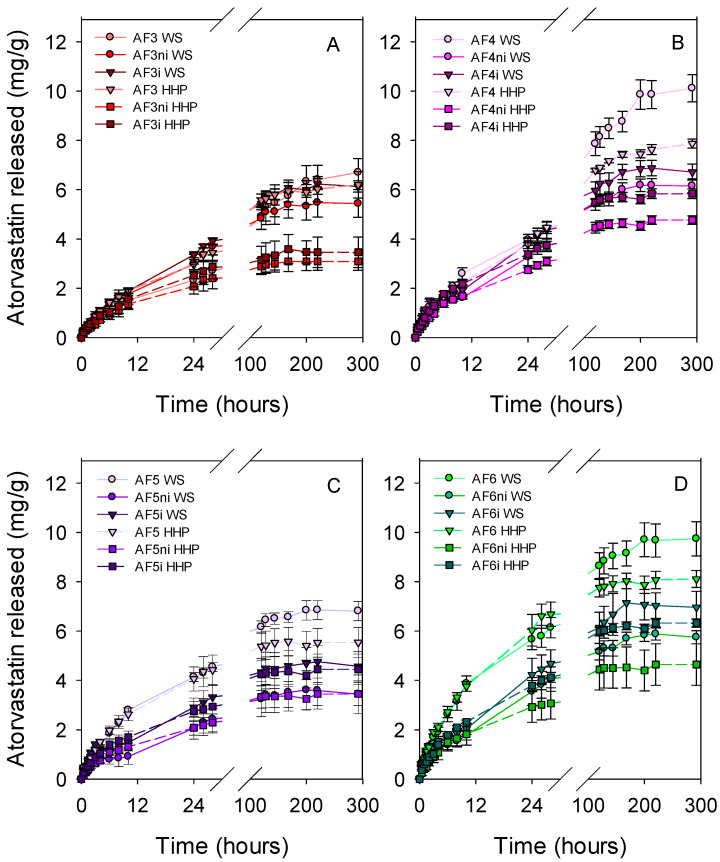
Comparison of atorvastatin release profiles from hydrogels that had not been sterilized (WS) and hydrogels that had been sterilized by high hydrostatic pressure (HHP) before loading. (**A**) F3 series, (**B**) F4 series, (**C**) F5 series, and (**D**) F6 series. Codes as in Table 1 (*n* = 4; mean values and standard deviations).

Then, hydrogels sterilized applying gamma radiation, steam heat, or HHP were tested again in terms of atorvastatin loading and release under the same conditions as for the nonsterilized hydrogels. Steam heat and HHP sterilized hydrogels evidenced a small decrease in the capability to load atorvastatin (Figure 8). This finding was especially remarkable for nonimprinted and imprinted hydrogels prepared by combining the two functional monomers EGPEM and AEMA (AF3ni and AF3i). This effect may be attributed to the impact of the moist heat conditions on the amino and hydroxyl groups that interact with the drug by hydrogen bonds, although no changes were recorded in the FTIR spectra probably because of the relatively low molar content in functional monomers, compared to pHEMA. Similar results were observed in previous studies using acrylic hydrogels containing 80–90% of polymethyl methacrylate (PMMA) and 10–20% of HEMA [43,78], in which steam heat sterilization decreased the amount of ketorolac and diclofenac loaded after 14 days of loading. As expected, the amount of drug released by the sterilized discs was lower, compared to nonsterilized hydrogels (Figure 9 and Figure S7 in Supplementary Materials).

After gamma-radiation sterilization, the amount of drug loaded by the hydrogels was remarkably low (below 1 mg/g of hydrogel, data not shown), and the amount of drug released was below the quantification limit. For this reason, and because gamma radiation triggered the degradation of the drug in an aqueous solution, this technique was discarded for further experiments.

**Figure 10:**
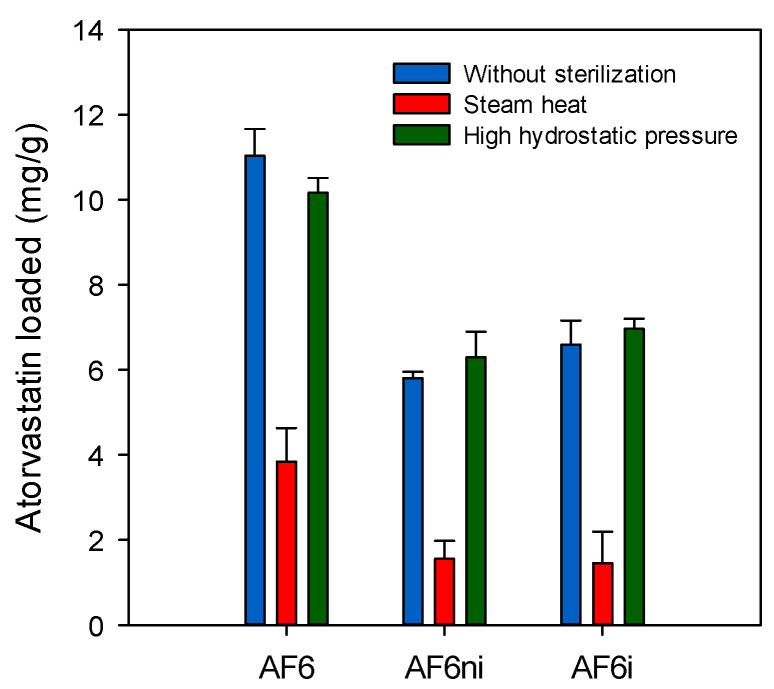
Atorvastatin loaded by AF6, AF6ni, and AF6i hydrogels when they were sterilized in drug solution by means of steam heat or high hydrostatic pressure, compared to nonsterilized hydrogels.

**Figure 11:**
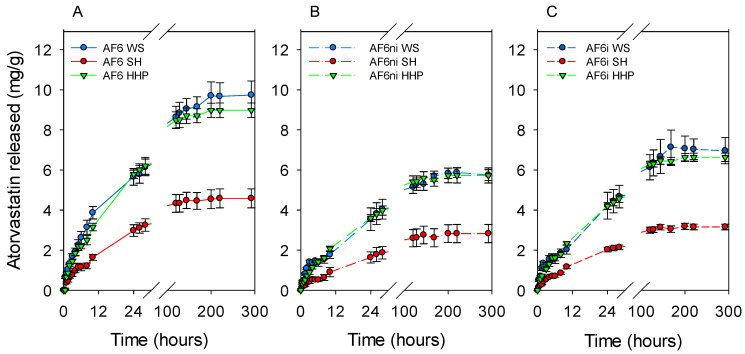
Comparison of atorvastatin release profiles in SLF from AF6 (**A**), AF6ni (**B**), and AF6i (**C**) hydrogels that had not been sterilized (WS) or that were sterilized during loading (immersed in drug solution) by means of steam heat (SH) or high hydrostatic pressure (HHP). Codes as in Table 1 (*n* = 4; mean values and standard deviations).

The next step was to elucidate the feasibility of performing the sterilization by steam heat or HHP while the hydrogels were already in the atorvastatin loading solution (one-pot processing). This procedure could allow for loading and sterilization in one step, which may be advantageous in terms of time and processing steps. Only the most promising hydrogels in terms of atorvastatin affinity and network stability, i.e., those prepared with APMA as a functional monomer (AF6, AF6ni, AF6i) were tested. The hydrogels were immersed in atorvastatin solution (0.04 mg/mL ethanol:water 20:80 *v/v*) for 48 h under magnetic stirring (150 rpm) at room temperature before steam heat or HHP sterilization. The hydrogels sterilized with steam heat loaded less drug than the nonsterilized hydrogels (Figure 10), which could be due to a combination of changes in the functional groups that interact with atorvastatin and degradation of the drug. Differently, the hydrogels that underwent HHP loaded similar amounts of atorvastatin as the nonsterilized ones. Namely, HHP sterilization did not affect the loading of atorvastatin. As expected, the amount of atorvastatin released from hydrogels sterilized with steam heat decreased, compared with nonsterilized hydrogels, while sterilization by HHP did not modify the drug released pattern (Figure 11). These results agreed well with those recorded for hydrogels that were first sterilized and then loaded with atorvastatin, suggesting that degradation of both the hydrogels and the drug under steam heat conditions may compromise the performance of the hydrogels as drug-eluting contact lenses.

### 3.6. Cornea and Sclera Permeability and Accumulation

Ex vivo permeability tests were carried out with the most promising hydrogels (AF6, AF6ni, and AF6i) to evaluate their capability of providing therapeutic amounts of atorvastatin to the eye structures. The amount of atorvastatin released from the hydrogels towards the donor chamber (filled with simulated lachrymal fluid) and then diffused toward cornea and sclera tissues, and the receptor medium was monitored by HPLC. An atorvastatin solution (60 µg/mL in ethanol:water 20:80 *v/v*, 1 mL) was also used as a control. The tests were performed using cornea and sclera tissues from porcine cattle since the porcine eyes are reported to present the most similar anatomical properties to the human eyes [79].

**Figure 12:**
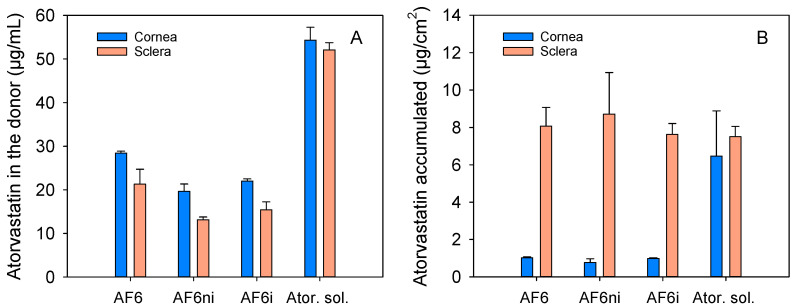
Amounts of atorvastatin in the donor chamber (**A**) and accumulated in cornea and sclera tissues (**B**) after 6 h of contact with drug-loaded hydrogels (AF6, AF6ni, and AF6i) and atorvastatin solution (60 µg/mL in ethanol:water 20:80 *v/v*, 1 mL). Codes as in Table 1 (*n* = 3; mean values and standard deviations).

Regarding the concentration of atorvastatin in the donor chamber after 6 h of experiment, these values were 28.43 ± 0.43 µg/mL for AF6, 19.67 ± 1.68 µg/mL for AF6ni and 22.00 ± 0.51 µg/mL for AF6i for cornea tissue (Figure 12A). For scleral tissue, the concentration of drug in the donor chamber was significantly lower for all hydrogels (21.32 ± 3.40 µg/mL for AF6; 13.17 ± 0.63 µg/mL for AF6ni; and 15.46 ± 1.81 µg/mL for AF6i; ANOVA, *p* < 0.05). In the case of the control solution, the concentration of atorvastatin remaining in the donor chamber was still significantly high in contact with cornea and sclera tissues (54.31 ± 2.98 and 52.11 ± 0.64 µg/mL, respectively) and, in all cases, greater than that recorded for the hydrogels (ANOVA, *p* < 0.05).

Amounts accumulated in cornea were 1.03 ± 0.04 µg/cm^2^ for AF6, 0.78 ± 0.19 µg/cm^2^ for AF6ni and 0.99 ± 0.04 µg/cm^2^ for AF6i (Figure 12B). These values were statistically different from those obtained for the atorvastatin solution (6.47 ± 0.41 µg/cm^2^, ANOVA, *p* < 0.05). In sclera, atorvastatin accumulation was higher and no statistical differences were observed between the drug-loaded hydrogels and the control drug solution (ANOVA, *p* = 0.78).

The amount of atorvastatin in the receptor chambers was below the quantification limit in the time frame of the study. This means that atorvastatin mainly accumulated into the cornea and sclera tissues but did not progress further or the diffusion was too slow to be detected.

In a reported preclinical study, atorvastatin calcium formulated as eye drops (50 µM drug concentration) was instilled 1 drop 8 times a day for 4 weeks in patients with blepharitis and dry eye, showing tear film stabilization and anti-inflammatory effects [16]. Therefore, in each instillation, roughly 0.6 µg drug dose was applied onto the cornea, and this amount could rapidly vanish due to lachrymal fluid turnover. The hydrogels designed in the present work provided amounts of atorvastatin accumulated in the cornea and sclera that may be considered therapeutically effective.

### 3.7. Stability of Atorvastatin Calcium Solution

Finally, since during CL wearing the hydrogels are exposed to daylight, drug stability against UV–Vis radiation may be critical for the successful development of a drug-eluting CL. Atorvastatin in solution was exposed to white light (fluorescent lamp) mimicking the daylight conditions. After 8 h of exposure, UV–Vis spectrum recorded by spectrophotometry and HPLC runs (Figure S8 in Supplementary Materials) of atorvastatin solutions before and after white light exposition were identical; this means that atorvastatin calcium did not suffer any degradation. Therefore, atorvastatin loaded by the designed hydrogels is expected to remain stable under daylight conditions, as also found in previous studies [80].

## 4. Conclusions

In the present work, bioinspired contact lenses were designed, for the first time, to have an increased affinity for atorvastatin calcium by mimicking the active site of HMG–CoA reductase. The synthesized hydrogels presented solvent uptake, light transmission, and mechanical properties values in the common range for commercially available CLs. No potential eye (CAM) irritation was observed neither cytotoxicity effects in Balb/3T3 cells. The APMA hydrogels are the most promising candidates to be used as drug-eluting contact lenses in terms of the amount of drug loaded and released. From the fabrication point of view, loading and sterilization in the same container (one-pot processing) is a crucial step. The loading capacity of the hydrogels and stability of atorvastatin was not affected by HHP sterilization, which may advantageously substitute steam heat and gamma radiation in the sterilization of atorvastatin-loaded hydrogels, maintaining atorvastatin stability and hydrogels performance. Permeability studies through the porcine cornea and sclera tissues revealed that the amount of atorvastatin accumulated in the cornea and sclera could be effective to treat ocular surface diseases. The success of this first attempt to design atorvastatin-loaded contact lenses must be contrasted with preclinical studies that confirm their efficacy in the treatment of ocular surface diseases.

## Figures and Tables

**Table 1:** Composition of the monomer mixtures used to synthesize the hydrogels. ni: nonimprinted; i: imprinted.

Hydrogel Code	HEMA (mL)	EGDMA (µL)	EGPEM (µL)	AEMA (mg)	APMA (mg)	Atorvastatin (mg)	AIBN (mg)
AF1	3	12.10	-	-	-	-	4.93
AF1ni	3	12.10	-	-	-	-	4.93
AF1i	3	12.10	-	-	-	12.57	4.93
AF2	3	12.10	112.50	-	-	-	4.93
AF2ni	3	12.10	17.18	-	-	-	4.93
AF2i	3	12.10	17.18	-	-	12.57	4.93
AF3	3	12.10	112.50	19.90	-	-	4.93
AF3ni	3	12.10	17.18	14.91	-	-	4.93
AF3i	3	12.10	17.18	14.91	-	12.57	4.93
AF4	3	12.10	112.50	-	21.45	-	4.93
AF4ni	3	12.10	17.18	-	16.08	-	4.93
AF4i	3	12.10	17.18	-	16.08	12.57	4.93
AF5	3	12.10	-	19.90	-	-	4.93
AF5ni	3	12.10	-	14.91	-	-	4.93
AF5i	3	12.10	-	14.91	-	12.57	4.93
AF6	3	12.10	-	-	21.45	-	4.93
AF6ni	3	12.10	-	-	16.08	-	4.93
AF6i	3	12.10	-	-	16.08	12.57	4.93

**Table 2:** Amounts of atorvastatin loaded by each hydrogel, and the K_N/W_ values calculated (mean value and in parenthesis standard deviations). Codes as in Table 1.

Hydrogel Code	Amount of Atorvastatin Loaded (mg/g)	K_N/W_
AF1	0.11 (0.08)	2.1 (2.3)
AF1ni	0.17 (0.20)	3.9 (6.2)
AF1i	0.18 (0.21)	4.1 (6.3)
AF2	0.09 (0.04)	1.7 (1.1)
AF2ni	0.16 (0.21)	3.4 (6.3)
AF2i	0.16 (0.21)	3.4 (5.6)
AF3	7.12 (0.38)	177.6 (11.6)
AF3ni	5.66 (0.12)	140.9 (3.0)
AF3i	5.22 (0.69)	130.1 (17.1)
AF4	11.32 (0.18)	282.6 (5.5)
AF4ni	6.31 (0.21)	157.2 (6.5)
AF4i	6.77 (0.27)	168.7 (6.6)
AF5	7.50 (0.28)	187.0 (8.6)
AF5ni	4.69 (0.35)	116.7 (10.7)
AF5i	5.00 (0.15)	124.6 (4.7)
AF6	11.08 (0.63)	276.4 (19.2)
AF6ni	5.81 (0.12)	144.7 (3.5)
AF6i	6.69 (0.23)	166.7 (7.0)

## Data Availability

Raw data are available upon request.

## Supplementary Materials

The following are available online at https://www.mdpi.com/article/10.3390/pharmaceutics13050606/s1, Table S1. Fitting of Higuchi equation; Figure S1. Light transmittance (%) of the hydrogels after being swollen in SLF and in drug solution; Figure S2. Atorvastatin release profiles (%) for AF3, AF4, AF5 and AF6 hydrogels in simulated lachrymal fluid under magnetic stirring; Figure S3. Pictures of HET–CAM test showing the CAM after 5 min in contact with atorvastatin-loaded discs and drug solution (0.04 mg/mL) in ethanol:water 20:80 *v/v*; Figure S4. FTIR–ATR spectra obtained for nonsterilized AF6, AF6ni, and AF6i hydrogels hydrated in NaCl 0.9% and sterilized with steam heat and HHP, Figure S5. UV-Vis spectra of atorvastatin calcium (0.04 mg/mL) solution in ethanol:water 20:80 *v/v* before and after steam heat, gamma-radiation and high hydrostatic pressure sterilization; Figure S6. HPLC chromatograms of the atorvastatin loading solution before and after sterilization processing applying steam heat and high hydrostatic pressure; Figure S7. Comparison of atorvastatin release profiles from hydrogels that had not been sterilized (WS) and hydrogels that had been sterilized by steam heat (SH) before loading; Figure S8. HPLC chromatogram of atorvastatin calcium solution (0.04 mg/mL in ethanol:water 20:80 *v/v*) after 8 h of exposure to white light.
