# Supplementary material for: Atorvastatin-Eluting Contact Lenses: Effects of Molecular Imprinting and Sterilization on Drug Loading and Release
Source: Pharmaceutics. 2021 Apr 22;13(5):606. doi: 10.3390/pharmaceutics13050606 (PMC8143582; doi:10.3390/pharmaceutics13050606)
Supplement: Supplementary file 1 [file pharmaceutics-13-00606-s001.zip › pharmaceutics-1170570-supplementary.pdf]

pharmaceutics

MDPI

Supplementary Materials: Atorvastatin-Eluting Contact Lenses: Effects of Molecular Imprinting and Sterilization on Drug Loading and Release

Ana F. Pereira-da-Mota, María Vivero-Lopez, Ana Topete, Ana Paula Serro, Angel Concheiro, and Carmen Al-varez-Lorenzo

Table S1. Fitting of Higuchi equation* Amounts of atorvastatin loaded by each hydrogel, and the $K_{N/W}$ values calculated (mean value and in parenthesis standard deviations). Codes as in Table 1.

Hydrogel code	Non-imprinted		Hydrogel code	Imprinted	
	k_H (%·h$^{-0.5}$)	R^2		k_H (%·h$^{-0.5}$)	R^2
AF3ni	11.77 (1.08)	>0.98	AF3i	12.43 (0.18)	>0.99
AF4ni	10.94 (0.93)	>0.98	AF4i	11.80 (1.02)	>0.98
AF5ni	9.46 (1.29)	>0.95	AF5i	11.24 (1.06)	>0.96
AF6ni	12.21 (1.59)	>0.97	AF6i	12.40 (1.03)	>0.97

*Higuchi equation was fitted to the release profiles in the 10% to 60% amount released interval as follows: $(Q/Q_0) *100 = k_H \cdot t^{0.5}$. In this Equation, Q represents the amount released at time t, Q_0 is the total amount loaded, k_H is the release rate constant, and the time t was expressed as hours.

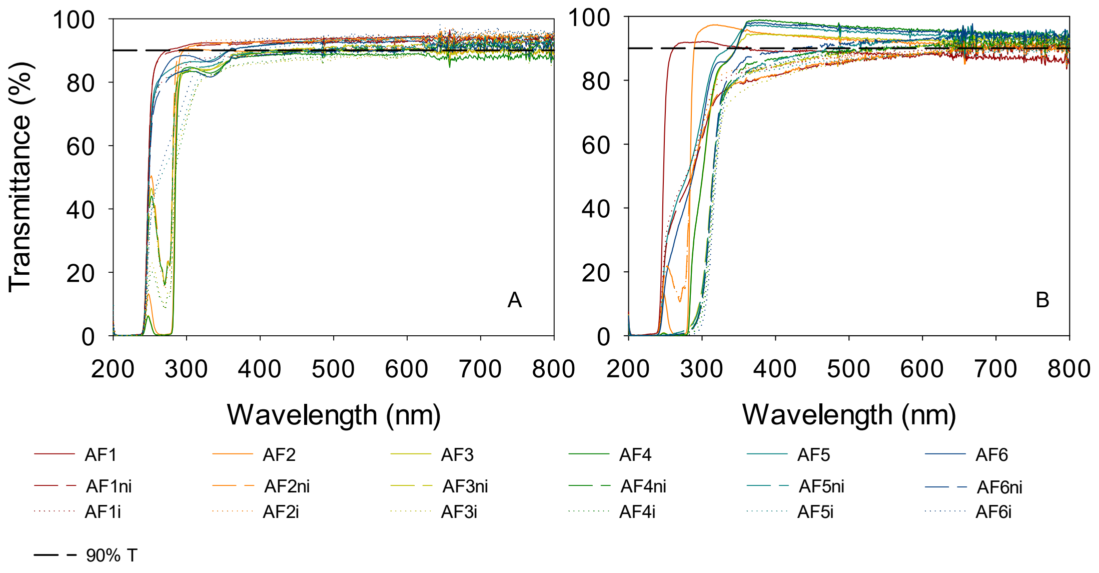

Figure S1. Light transmittance (%) of the hydrogels after being swollen in SLF (**A**) and in drug solution (**B**).

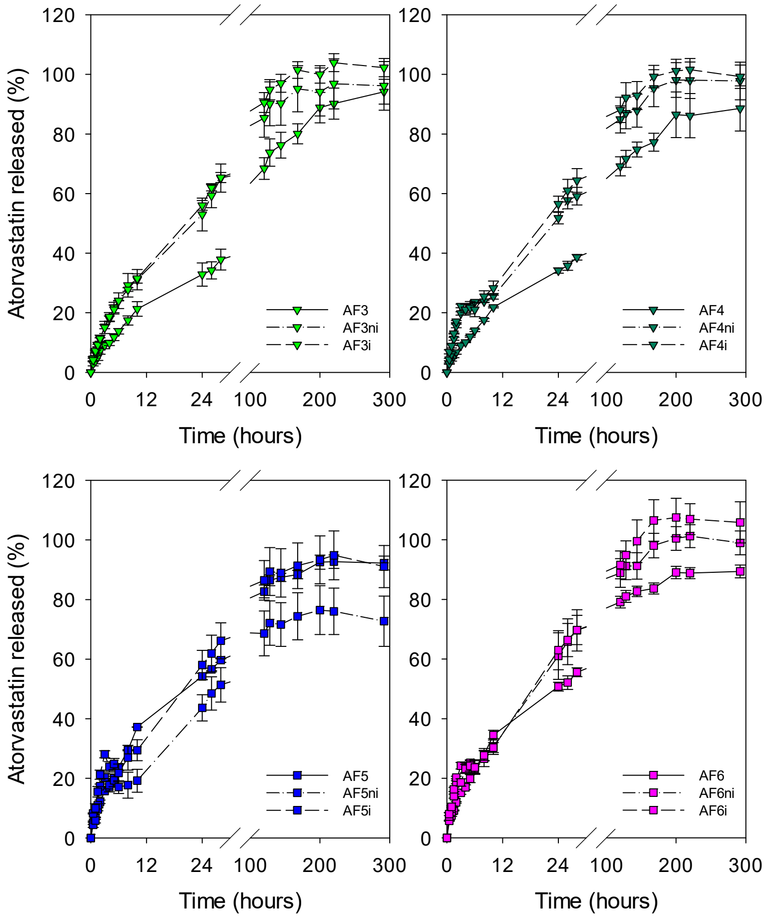

Figure S2. Atorvastatin release profiles (%) for AF3, AF4, AF5 and AF6 hydrogels in simulated lachrymal fluid at 37 ºC under magnetic stirring. Codes as in Table 1 (n=4; mean values and standard deviation).

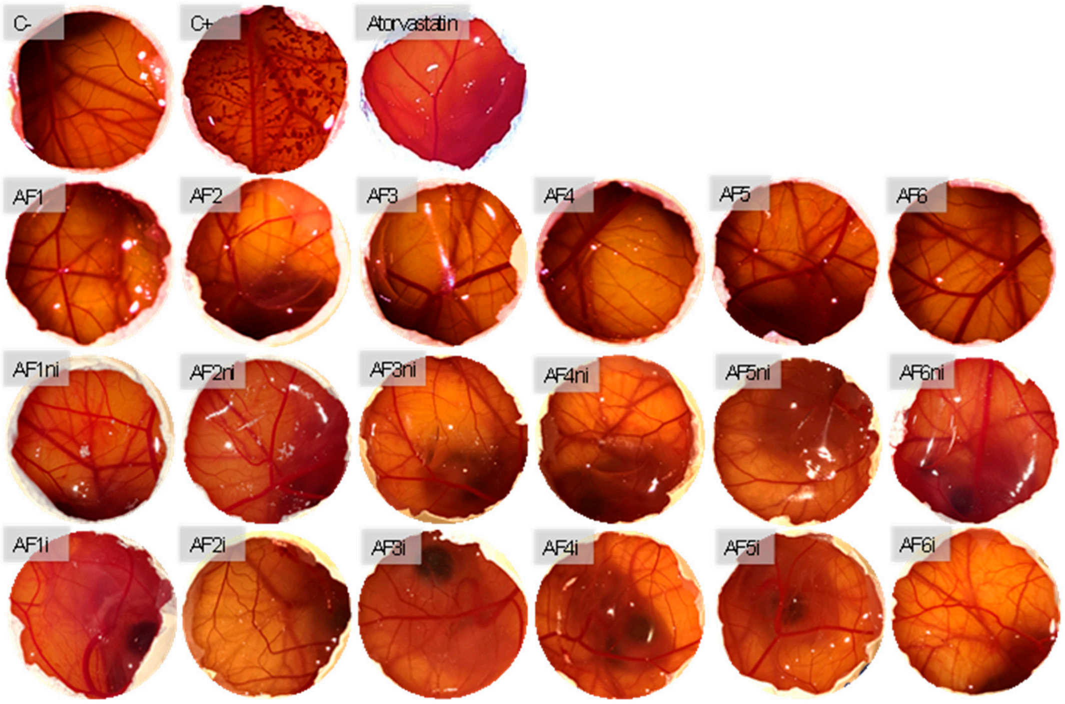

Figure S3. Pictures of HET-CAM test showing the CAM after 5 min in contact with atorvastatin-loaded discs and drug solution (0.04 mg/mL) in ethanol:water 20:80 *v*/*v*. Effects of negative (0.9% NaCl) and positive (0.1 N NaOH) controls are also shown. The diameter of the HET-CAM portion showed was 2 cm.

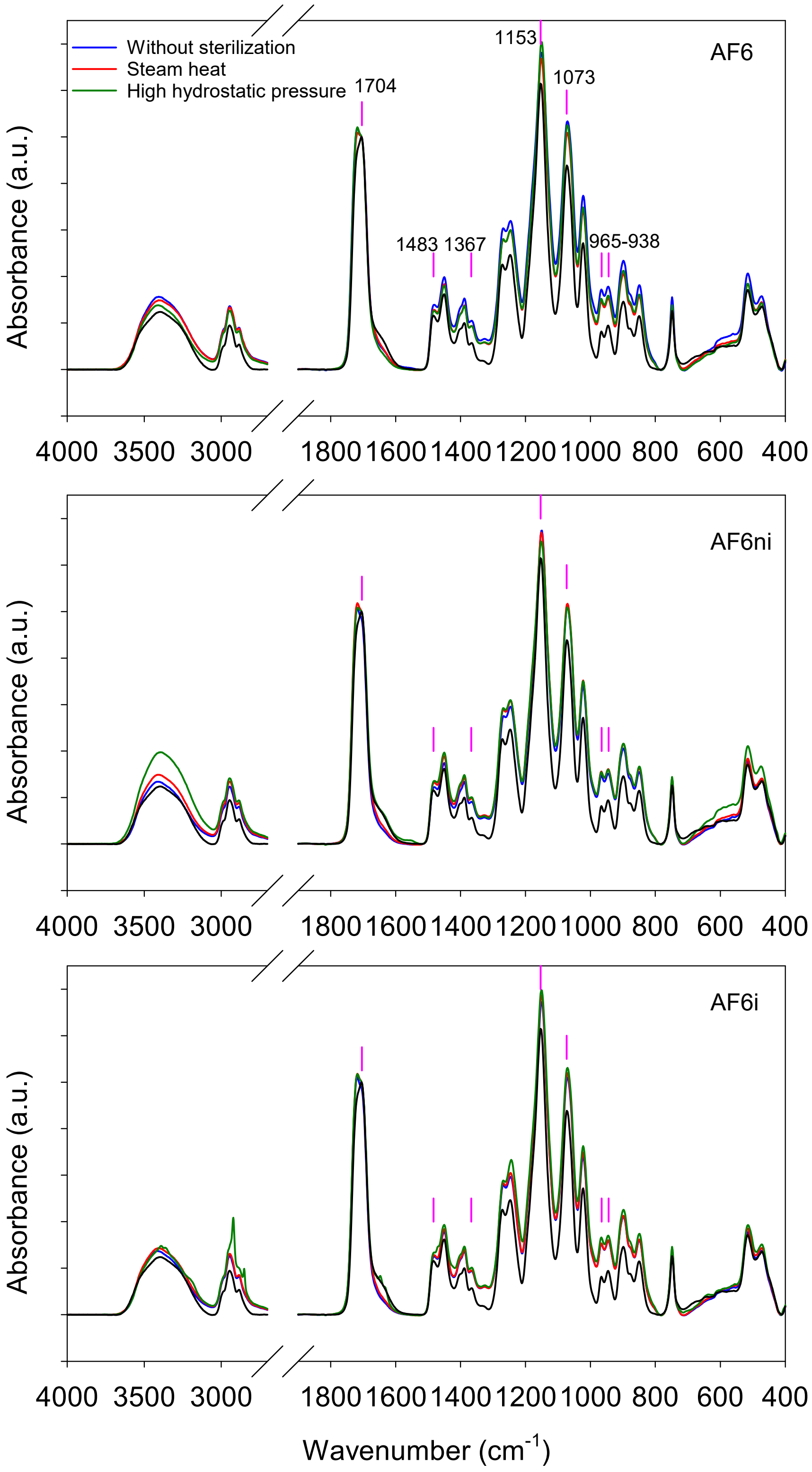

Figure S4. FTIR-ATR spectra obtained for AF6, AF6ni and AF6i hydrogels hydrated in NaCl 0.9% before and after being sterilized with steam heat and HHP. As a reference AF1 hydrogel spectrum is shown in black in the three plots.

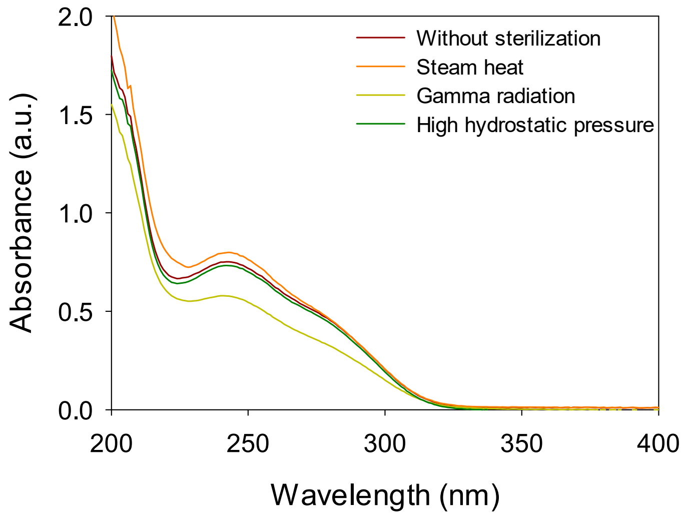

Figure S5. UV-Vis spectra of atorvastatin calcium (0.04 mg/mL) solution in ethanol:water 20:80 *v/v* before and after steam heat, gamma-radiation and high hydrostatic pressure sterilization.

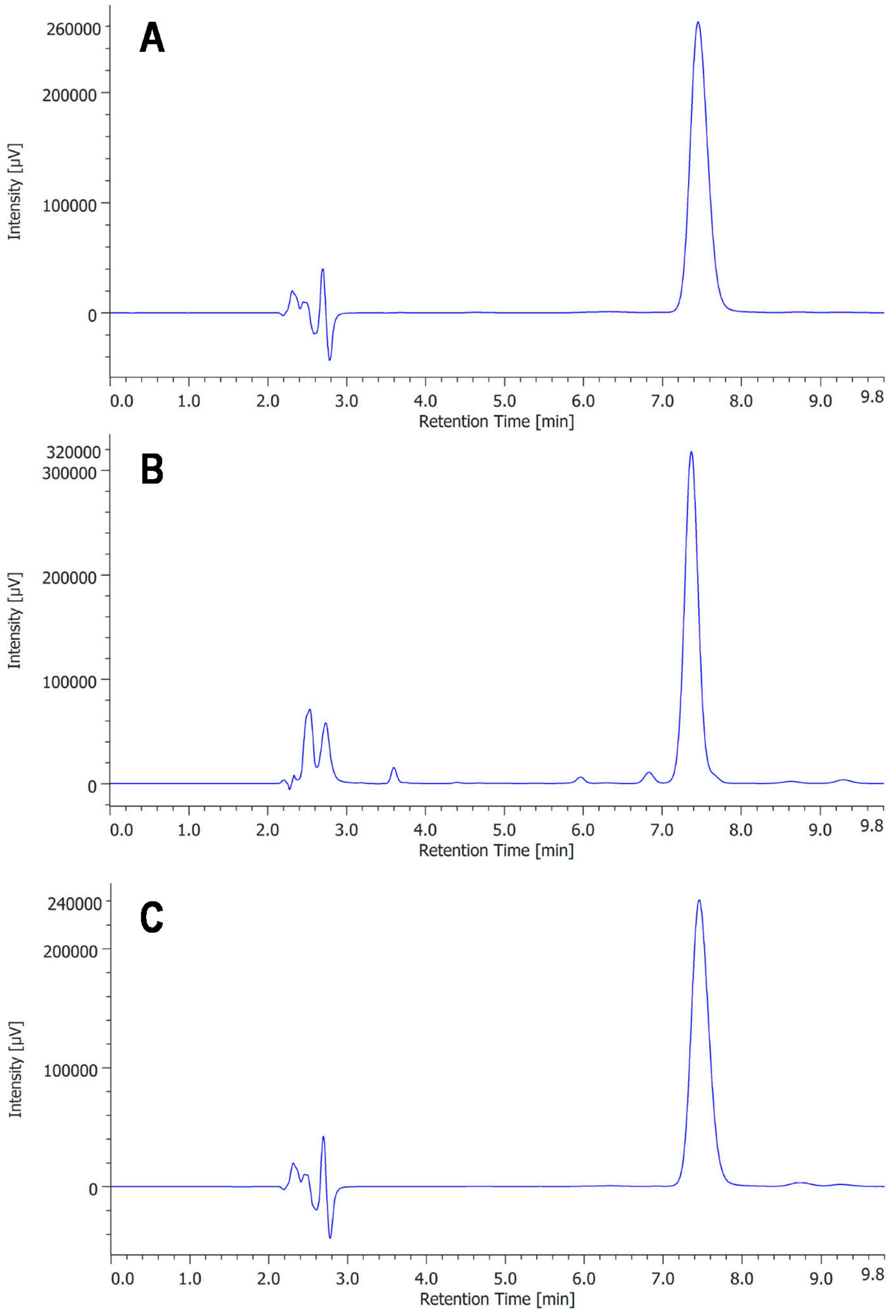

Figure S6. HPLC chromatograms of the atorvastatin loading solution before (**A**) and after sterilization processing applying steam heat (**B**) and high hydrostatic pressure (**C**).

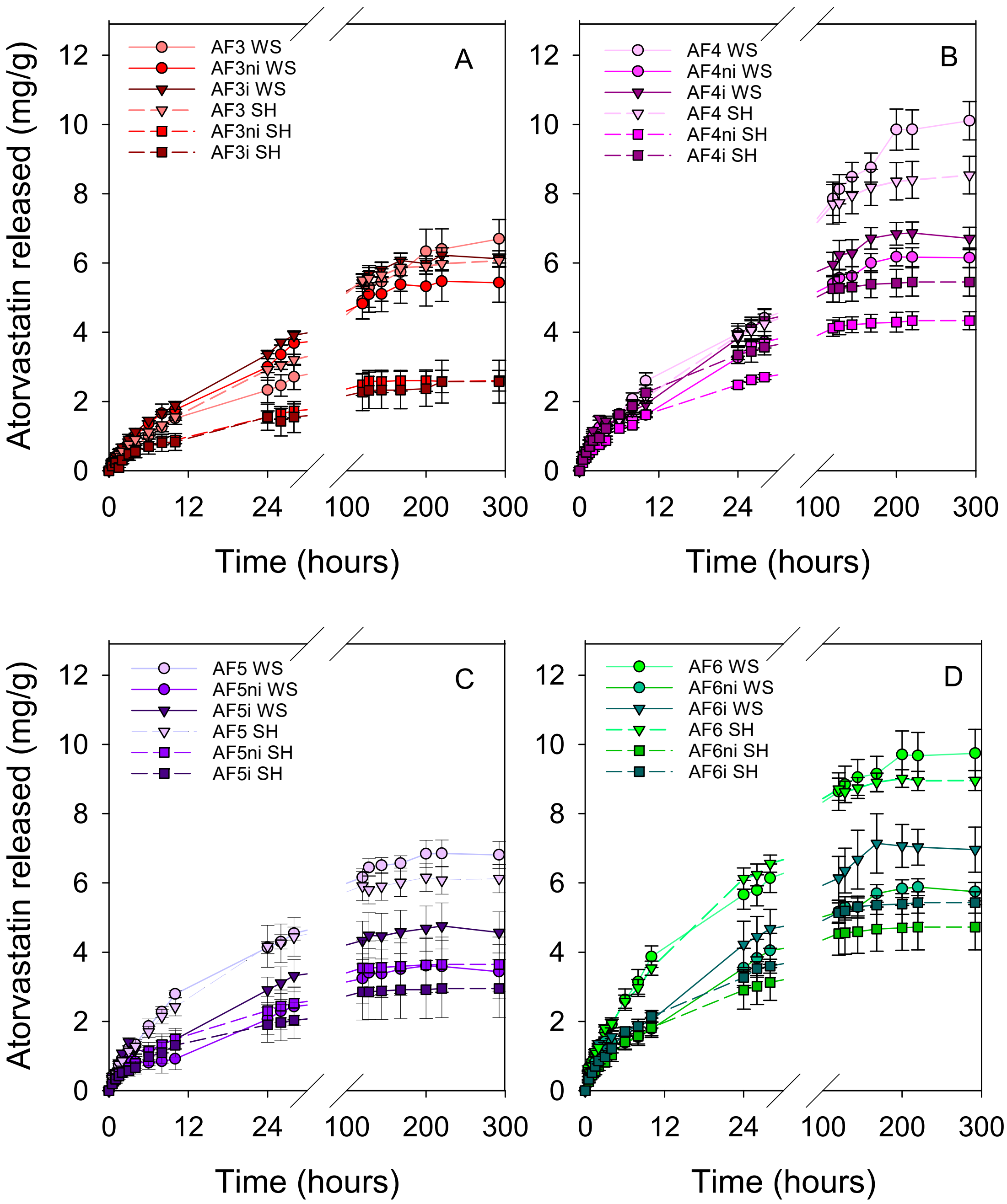

Figure S7. Comparison of atorvastatin release profiles from hydrogels that had not been sterilized (WS) and hydrogels that had been sterilized by steam heat (SH) before loading. (A) F3 series, (B) F4 series, (C) F5 series, and (D) F6 series. Codes as in Table 1 (*n*=4; mean values and standard deviations).

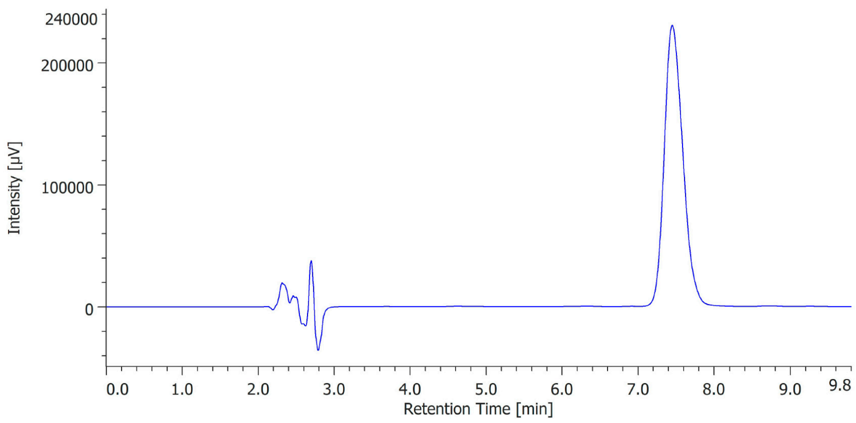

Figure S8. HPLC chromatogram of atorvastatin calcium solution (0.04 mg/mL in ethanol:water 20:80 *v*/*v*) after 8 h of exposure to white light.
